# Multispectral in-line hologram reconstruction with aberration compensation applied to Gram-stained bacteria microscopy

**DOI:** 10.1038/s41598-023-41079-4

**Published:** 2023-09-02

**Authors:** Dylan Brault, Thomas Olivier, Nicolas Faure, Sophie Dixneuf, Chloé Kolytcheff, Elodie Charmette, Ferréol Soulez, Corinne Fournier

**Affiliations:** 1 Université Jean Monnet Saint-Etienne, CNRS, Institut d Optique Graduate School, Laboratoire Hubert Curien UMR 5516, 42023 Saint-Etienne, France; 2 bioMérieux, Centre Christophe Mérieux, 38024 Grenoble, France; 3 BIOASTER, Bioassays, Microsystems and Optical Engineering Unit, Lyon, France; 4 bioMérieux, 376 Chemin de l’Orme, 69280 Marcy-l’Etoile, France; 5 Univ. de Lyon, Université Lyon1, ENS de Lyon, CNRS, Centre de Recherche Astrophysique de Lyon, UMR 5574, 69230 Saint-Genis-Laval, France

**Keywords:** Computational science, Interference microscopy

## Abstract

In multispectral digital in-line holographic microscopy (DIHM), aberrations of the optical system affect the repeatability of the reconstruction of transmittance, phase and morphology of the objects of interest. Here we address this issue first by model fitting calibration using transparent beads inserted in the sample. This step estimates the aberrations of the optical system as a function of the lateral position in the field of view and at each wavelength. Second, we use a regularized inverse problem approach (IPA) to reconstruct the transmittance and phase of objects of interest. Our method accounts for shift-variant chromatic and geometrical aberrations in the forward model. The multi-wavelength holograms are jointly reconstructed by favouring the colocalization of the object edges. The method is applied to the case of bacteria imaging in Gram-stained blood smears. It shows our methodology evaluates aberrations with good repeatability. This improves the repeatability of the reconstructions and delivers more contrasted spectral signatures in transmittance and phase, which could benefit applications of microscopy, such as the analysis and classification of stained bacteria.

## Introduction

Quantitative phase imaging (QPI) is one of the modalities that extended the range of microscopy in the biomedical field^1^. Indeed, this modality offers more than just morphological parameters: it can provide interesting biological insights, for example, into dry mass^2^, cell viability^3^ or cell life cycle characterization^4^. Moreover, its nanometric precision on the optical path difference (OPD) paves the way for the study of very weak phenomena at the microscopic level^5,6^. Different methods can be used to perform QPI: interferometry, wavefront sensing and digital holography^7^. All these methods have in common that the sensor records an intensity map in which the phase information is encoded in different ways. Retrieval of the phase thus requires a numerical reconstruction step. Moreover, whatever the method, the quantitative reconstruction of the phase is sensitive to focusing errors^7,8^ and to aberrations induced by different elements in the optical train^9–12^.

Concerning QPI’s sensitivity to focusing errors, methods that work with out-of-focus samples have a distinct advantage, as the focus position is numerically estimated during reconstruction. Among others, digital in-line holographic microscopy (DIHM) is a method based on this principle. From the experimental point of view, this configuration is the simplest as it only consists in defocused microscopy under coherent illumination. It is easy to set up, relatively cheap, very stable and does not require precise control of the focus position. The coherence length requirements are also low, meaning LED sources can be used instead of lasers. However, this holography configuration works under the hypothesis of sparse samples and reconstruction consists in a phase retrieval problem with a numerical refocusing step. A reconstruction based on a simple backpropagation step of the recorded intensity is known to lead to the twin-image artifact because the sign of the wavefront curvature on the sensor is lost in the intensity measurement. To address the twin-image problem, phase retrieval can be solved using alternating projection algorithms^13–15^ or regularized inverse problems approaches (IPA)^16,17^.

In the particular case of objects that can be parameterized with few variables, model-based approaches can also be efficiently used^18–20^. For example, DIHM holograms can be processed by fitting a Lorenz-Mie model to the diffraction patterns produced by spherical objects. In this case, the reconstruction problem can be solved more easily as the spherical geometry of the objects is imposed as the prior directly in the model. Note that for these objects, the reconstruction problem is equivalent to estimating their parameters. The advantages of this approach are that the twin-image artifact is no longer a problem, the thickness and the refractive index can be estimated separately and the accuracy on the parameters is not limited to the pixel size of the sensor. However, like any other QPI method, whatever the algorithm used to address the phase retrieval problem, it is still sensitive to focus biases due to aberrations of the optical system.

The use of high numerical aperture objectives is mandatory in microscopy applications that require the best possible optical resolution. In this case, the conventional depth of field is less than one micrometer and may thus be highly sensitive to aberrations. Even with a high grade aberration correction (apochromatic objective), in this work, we show that some residual aberrations can still significantly modify the on-axis and lateral positions of objects as a function of the wavelength (longitudinal and lateral chromatic aberrations). The diffracted patterns in the holograms are also significantly distorted by geometrical aberrations, leading to distortions and biases in the reconstructed complex transmittance^12^ or to biases in the estimation of the parameters with model-based analysis^11,12,21^. Note that, to our knowledge, the importance of this problem in DIHM has only quite recently been studied.

To address these issues, we use a 2-step unsupervised method. First, the calibration step consists in estimating the parameters of calibration beads previously inserted in the sample, as well as the aberration parameters at the same time. Our estimations use a modified Lorenz-Mie model that includes the effect of aberrations with the use of Zernike polynomials formalism^22,23^. Calibration is performed at various positions in the field of view and at each wavelength. Second, the estimated parameters (that may vary in the field of view and with the wavelength) are used to reconstruct an in-focus complex transmittance of the samples with a regularized inverse problem approach in which the propagation model includes the estimated aberrations. The multi-wavelength data are reconstructed jointly using a single regularization that promotes the colocalization of the object edges at every wavelength^24^. To this end, the IPA reconstruction uses phase diversity, which has been proven to be very efficient in lens-free configurations with the use of multi-height acquisitions^25^, multi-angle illuminations^26,27^ or multi-wavelength illuminations^24,28–30^. The novelty of this work is to estimate and take into account the geometrical and chromatic aberrations of the optical system to ensure the validity of the colocalization hypothesis and thus take full advantage of the multi-wavelength phase diversity.

**Figure 1:**
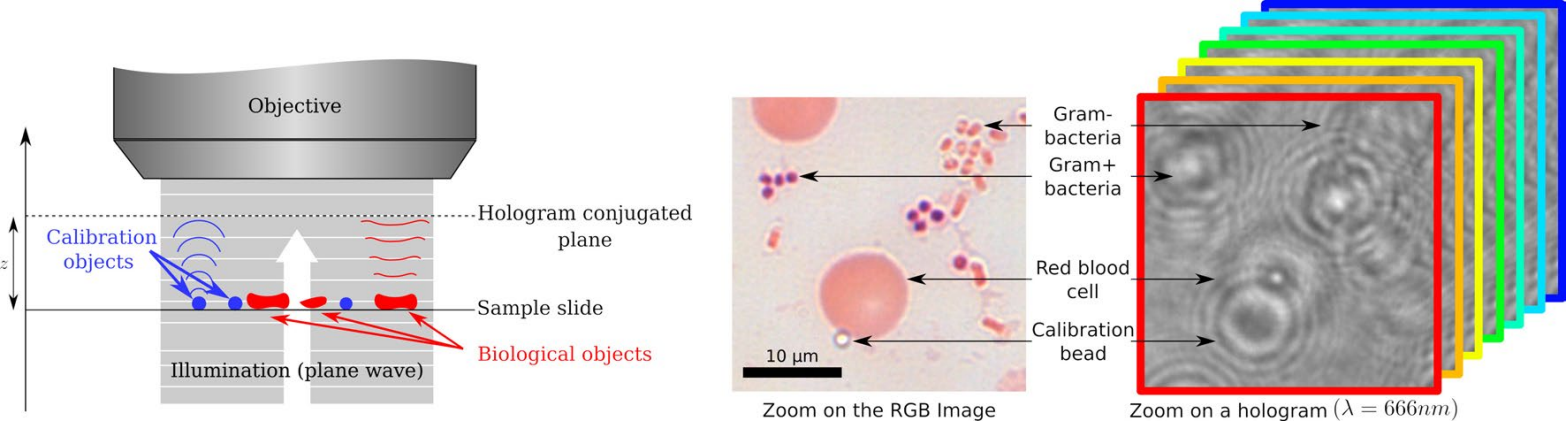
Experimental principle of our study: Left: experimental configuration of DIHM with plane wave illumination and defocused imaging of both calibration beads and biological objects of interest. Center: Example of a conventional color image of a Gram-stained blood smear showing red blood cells, Gram positive and Gram negative bacteria and calibration beads. Right: Corresponding diffraction patterns of these objects illustrated on the multispectral hologram stack.

We apply our reconstruction method to Gram-stained slides produced from bacteria infected blood smears. The experimental configuration, conventional images and holograms of the samples studied here are illustrated in Fig. 1. Such smears are routinely implemented at large scale in microbiology laboratories and require high-resolution and color microscopy. Errors in readings of such smears are not rare and can have a substantial impact on patient care^31,32^. The expertise required to read Gram smears is eroding in many laboratories^33^, driving a need for improved automation and standardization, but the current offer does not address this need^34^. In this context, providing a robust and quantitative measure will substantially improve the performances of automated systems. To do so in the context of stained samples, it is then interesting to consider the use of multi-wavelength reconstructions exploiting the phase diversity without any prior on the spectral properties of the samples. Thus, it allows to explore the spectral behavior of colored biological samples in transmission, but also in OPD, which gives an additional information on the sample. Also, using a quantitative phase imaging method helps reducing the imaging artifacts traditionally observed in standard white-light microscopy (aberrations, dispersion effects, halos, field varying focus, local focusing or defocusing of light by objects, etc.).

## Results

In this section, we present the results of our calibration and of the multispectral IPA reconstruction, which are detailed in the “Methods” section. The reconstructions were performed on an experimental dataset of holograms acquired with a home-made, automated microscope that is also described in the “Methods” section. The microscope recorded holograms of Gram-stained blood smears containing Gram positive and Gram negative bacteria and calibration silica beads at 8 different wavelengths. In this section, before describing the multispectral reconstructions on blood smears, we first present a critical analysis of the relevance of our aberrations calibration and the consistency and repeatability of these aberrations and focus position estimations.

### Chromatic aberrations calibration

This subsection describes the results of the calibration step. This calibration step estimates the phase of the amplitude transfer functions (ATF)^35^ of the optical system in coherent illumination. This ATF corresponds to the *generalized pupil function*, but with spatial frequency coordinates. Thus it is sometimes referred to as *pupil function*. The calibration is performed using calibration beads embedded in the sample. The aberration estimation uses a model-based approach that takes aberrations into account by modeling them with 14 Zernike polynomials in the exit pupil plane of the objective (see details in the “Methods” section).

**Figure 2:**
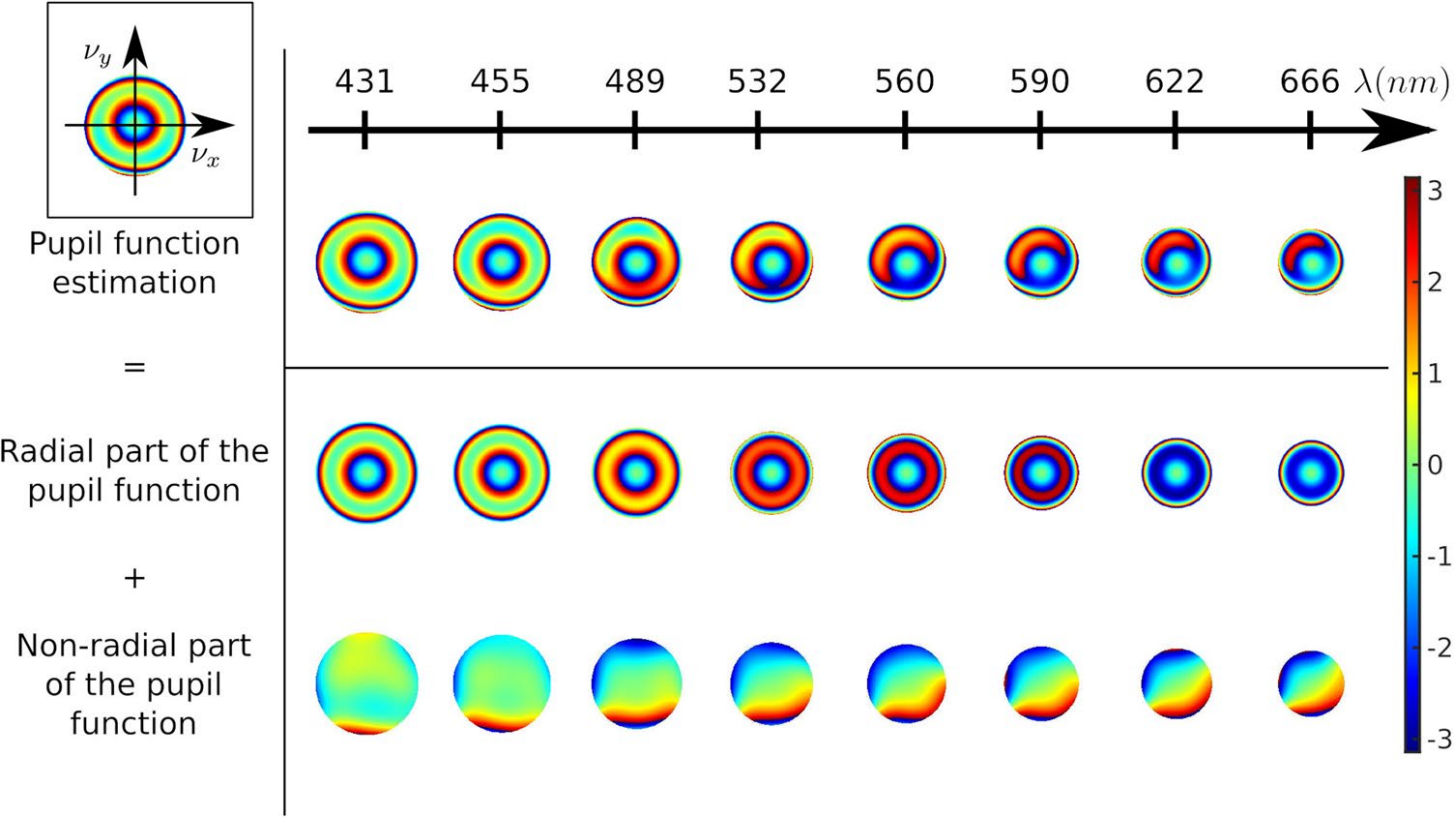
Examples of the pupil function estimated for the whole set of wavelengths. For easier visualization of the aberrations, the radial components (defocus and spherical aberrations) and non-radial components (tilt, coma, astigmatism, trefoil and quadrafoil) are presented separately at the bottom of the figure. In the top-left inset, $\nu _x$ and $\nu _y$ are the spatial frequencies in Fourier space.

Figure 2 illustrates the evolution of the estimated ATF as a function of the wavelength for one bead. As expected, this evolution is continuous, which seems physically consistent. For easier visualization of the continuity of the chromatic aberrations with the wavelength, a decomposition of the ATF as a sum of radial polynomials and non-radial polynomials is presented. The radial polynomial part corresponds to defocus and spherical aberrations and the non-radial part includes tilts, coma, astigmatism, trefoil and quadrafoil aberration components. Figure 2 shows that both these parts are continuous with the wavelength and non negligible. For example, from the non-radial part, we see that some tilt aberrations are present and vary with the wavelength. Indeed, these tilt aberrations correspond to phase ramps in the Fourier domain and lead to shifts in the spatial domain.

**Figure 3:**
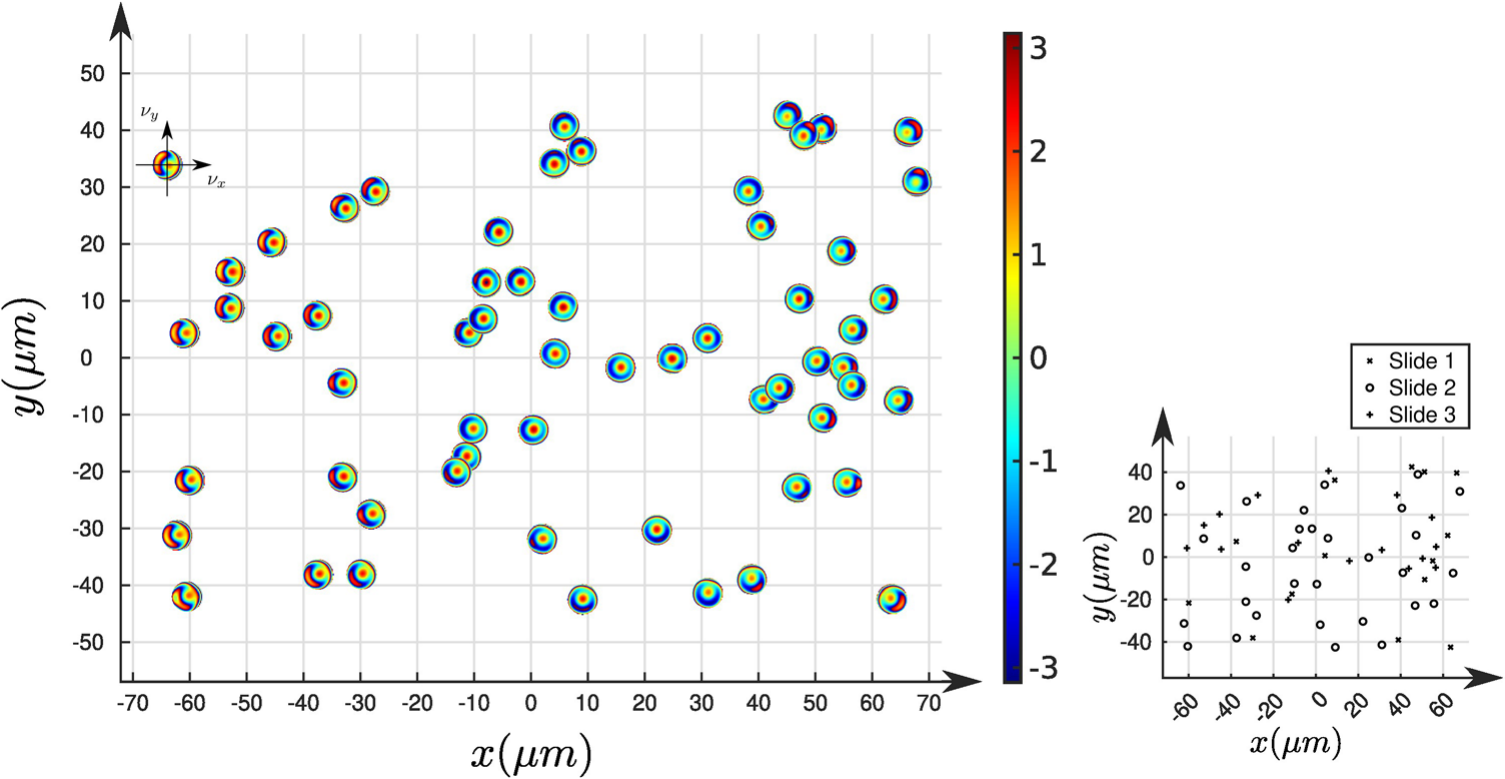
Left: Illustration of the evolution of the estimated ATFs in the field of view at $\lambda =532\,\textrm{nm}$. Right: Further details on the location (slide 1, 2 or 3) of the beads used in the ATF estimation.

Figure 3 illustrates the spatial evolution of the ATF at $\lambda =532\,\textrm{nm}$ in the field of view, at the location of the calibrated beads (more details on the Zernike coefficients are provided in Fig. S4). As expected, and without any prior in the estimations, the ATFs vary continuously and symmetrically in the field of view. Indeed, the non-radially symmetrical components of the aberrations (coma, astigmatism, etc.) appear to vanish near the center of the field. This kind of behavior is typical of an axisymmetric optical system, where some aberrations (like coma and astigmatism), are zero on the optical axis, but increase with the radial distance from the optical axis.

It should be noted that the ATFs in this illustration were estimated independently using 5 fields of view of 3 different slides, which do not change the continuity of the ATFs. This illustrates the repeatability of the proposed calibration from one field to the other and from one slide to the other. This repeatability shows that, in our experimental case, the aberrations come from the optical system alone, independently of the sample. It should, in this case, be possible to pre-calibrate the aberrations of the optical system with calibration beads alone and then using these estimations for the reconstruction of biological samples. Yet, inserting calibration beads in the sample is useful since it also provides an accurate estimation of the position of the slide plane^8^ that cannot be accomplished by a pre-calibration step.

### Hologram reconstructions and spectral analysis

#### Hologram reconstructions

**Figure 4:**
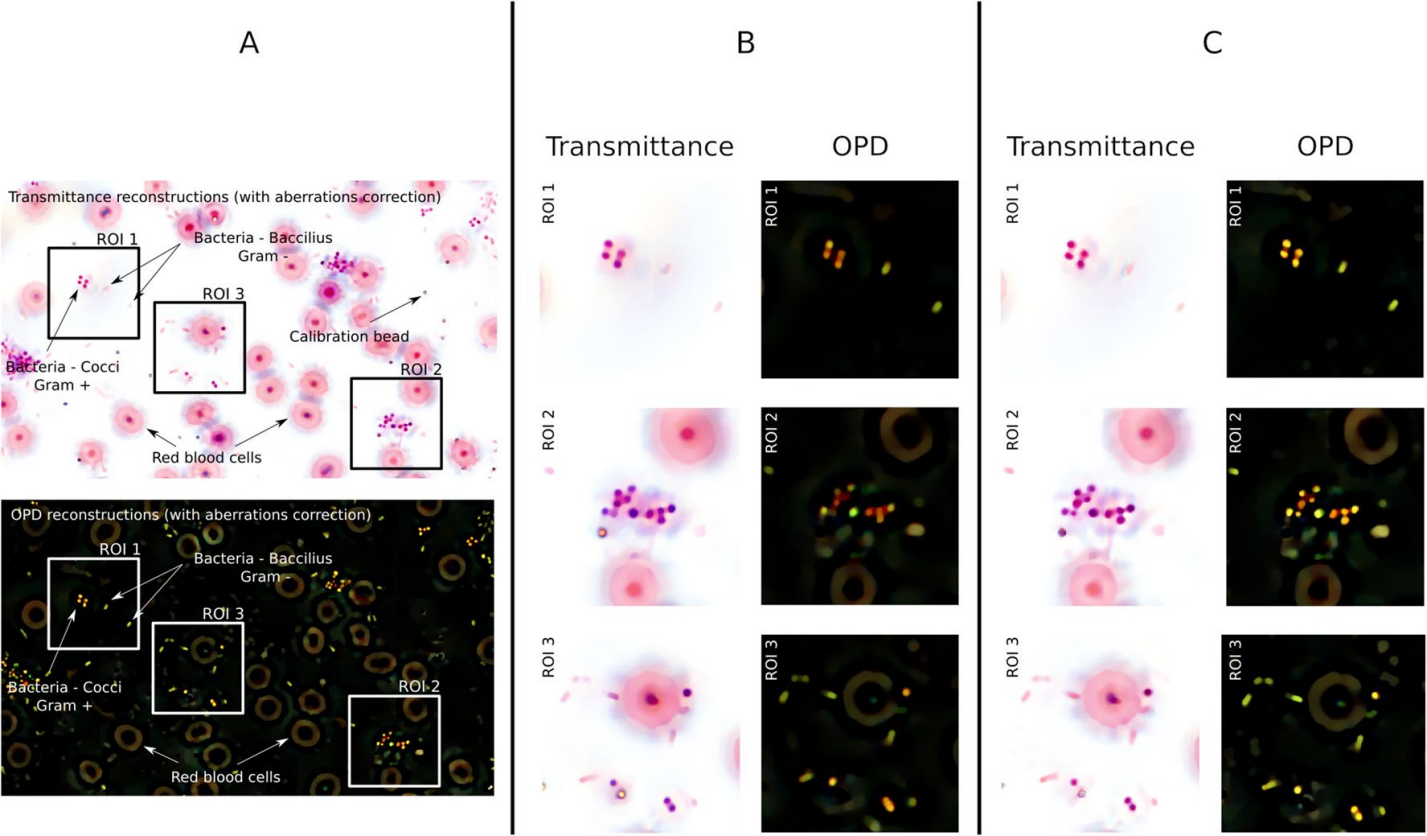
Illustration of transmittance and OPD color reconstructions of a sample containing two types of bacteria (Gram positive *Staphylococcus aureus* and Gram negative *Escherichia coli*). (**A**) Full field. (**B**) Regularized reconstructions reconstructed independently at each $\lambda$, with no correction of aberrations and at a fixed focus estimated at $431\,\textrm{nm}$. (**C**) Multi-wavelength joint reconstructions with aberration correction and with the colocalization prior.

After fitting a plane to the positions of the beads and interpolating the ATF in the field of view^36^, it is possible to perform accurate regularized reconstructions that fully exploit the multispectral information. As detailed in the “Methods” section, multispectral data are reconstructed using a prior that favours colocalized objects in the multiwavelength transmittance stack. For the purpose of display, we synthesized standard 3-channel color images from the reconstructed transmittance and optical path difference (OPD) maps at the 8 wavelengths, also described in the “Methods” section. Figure 4 illustrates the results of these regularized reconstructions using our multispectral aberration correction methodology (A,C). For the sake of comparison, Figure 4B presents a color reconstruction performed with a degraded version of our algorithm: First the aberrations were disregarded in both the calibration step and the reconstruction step; second, the reconstructions were performed independently at each wavelength, without the colocalization prior; and third, the focus estimation at $431 \textrm{nm}$ was kept constant for every wavelength. These two reconstruction approaches are hereafter referred to respectively, as *corrected reconstructions* and *uncorrected reconstructions*. The sample reconstructed in Figure 4 contains Gram positive cocci bacteria (*Staphylococcus aureus*) and Gram negative bacilli bacteria (*Escherichia coli*) as well as silica beads.

In Fig. 4, the corrected reconstructions show bacteria that are globally more contrasted, particularly in OPD reconstructions. This is clear in all the regions of interest (ROI) presented, but particularly in the OPD reconstruction of the clusters in ROI 1 and ROI 2. With or without correction, note that the Gram negative bacteria (light pink bacilli) are only weakly absorbing. They appear with a low contrast in the transmittance image and are much more visible in the OPD image. This is a good argument for the use of phase measurement on stained samples. However, the contrast enhancement does not seem that much important on the transmittance reconstructions, and is further analyzed and discussed in the “Spectral analysis” subsection below.

Another interesting improvement is that bacteria of the same type (in our example, either a Gram positive coccus or a Gram negative bacillus) are less dispersed in color in the corrected reconstructions, both in transmittance and OPD. This is particularly clear in the OPD of the cluster of bacteria in ROI 2. In the uncorrected reconstructions, the OPD colors of bacteria of the same type vary from red to green, whereas in the corrected reconstructions, the colors are more consistent, all roughly yellow.

Concerning the improved morphology, it can be seen that the bacteria are less blurry, both in transmittance and OPD on the corrected reconstructions. This improvement is easy to see in the clusters of ROI 1 and ROI 2, where the bacteria are not clearly separated with the uncorrected method. Once again, this is clearer on the ODP maps. In the uncorrected reconstructions in ROI 3, two adjacent cocci at the bottom are indistinguishable and look like a single bacillus, which is not the case on the corrected reconstructions.

**Figure 5:**
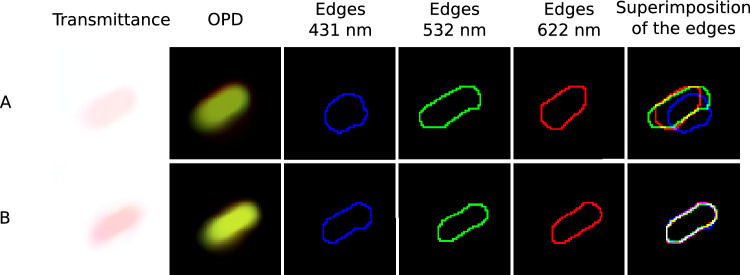
Illustration of the wavelength dependent lateral shifts and shape distortions of a bacterium (*Escherichia coli*) on uncorrected reconstructions (**A**) compared with our aberration correction methodology (**B**).

To further illustrate the morphological improvements enabled by chromatic aberration correction, Fig. 5 presents reconstructions zoomed on a single Gram negative bacillus. Reconstructions without aberration corrections (A) and reconstructions with aberrations corrections (B) are compared. Note that the focus has been corrected in both (A) and (B) to better highlight the lateral shifts and distortion effects of chromatic aberrations.

For three wavelengths in the blue (431 nm), green (532 nm) and red (622 nm) channels, the edges of the bacterium were computed using a watershed algorithm on the gradient of the reconstructed transmittance ${\varvec{t}}_\lambda$ evaluated with ${\varvec{G}}_{\lambda }=\sqrt{\Vert \nabla _k \Re ({\varvec{t}}_\lambda )\Vert ^2+\Vert \nabla _k \Im ({\varvec{t}}_\lambda )\Vert ^2}$, where $\nabla _k$ is a discrete gradient operator and $\Re$, $\Im$ the real and imaginary parts. Note that the gradient map is consistent with the regularization term used to incorporate colocalization in our multispectral reconstructions. The edges of bacteria presented in Fig. 5 thus illustrate the validity of the colocalization prior with or without aberration corrections. As expected, without aberration corrections (A), the edges are distorted and shifted differently at the three wavelengths, whereas with the aberration correction, they are perfectly superimposed. This shows that the chromatic aberrations significantly invalidate the colocalization prior of edges unless they are compensated for.

#### Spectral analysis

For the purpose of display, the reconstructed 8-channel transmittance and OPD maps were combined into RGB images in the previous figures. The full multispectral reconstructions make it possible to study the transmittance and OPD spectral signatures of biological organisms at the micrometer level, and with sixteen channels. For example, in the case of our Gram-stained samples, this spectral analysis could be used to improve classification of Gram-stained bacteria. In this subsection, we analyze the spectral signatures of four Gram-stained blood smears containing four types of bacteria: 2 Gram positive (*Staphylococcus aureus, Corynebacterium aurimucosum*) and 2 Gram negative (*Escherichia coli, Acinetobacter baumannii*). From the reconstructed images, a manual masking step was performed to isolate the pixels of roughly 100 individual bacteria of each type. Next, the spectral signatures in transmittance and OPD were extracted by averaging the pixel values of each type of bacteria. The dispersion was then evaluated by computing the standard deviation of the averaged values obtained using around 100 bacteria per type.

**Figure 6:**
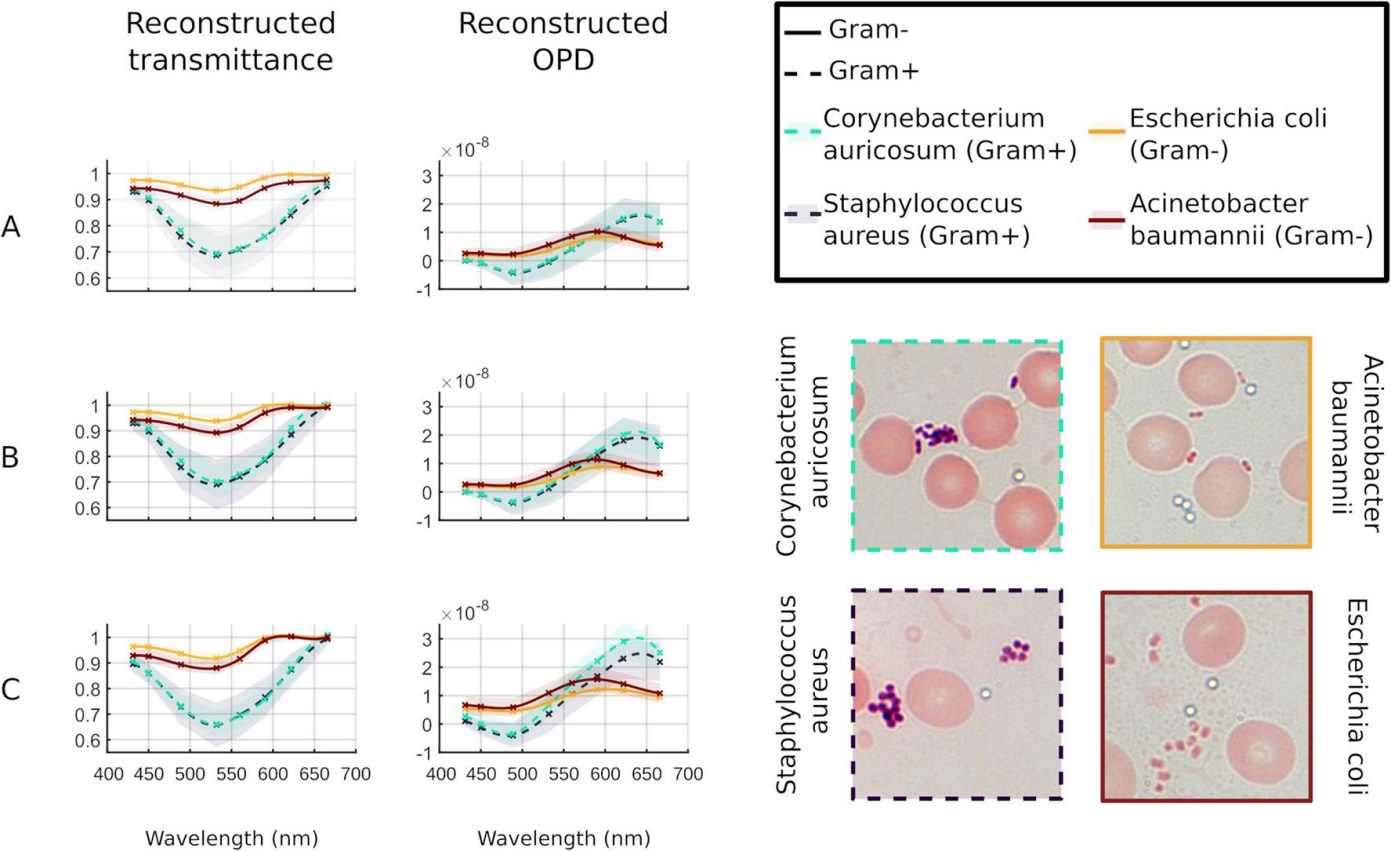
Illustrations of the spectral signatures in transmittance and OPD for 2 Gram positive and 2 Gram negative bacteria. (**A**) Uncorrected, independent reconstructions at each wavelength with a fixed focus evaluated at 431 nm. (**B**) Uncorrected, independent reconstructions with estimation of the focus at each wavelength. (**C**) Aberration corrected method with aberration corrected estimation of the focus at each wavelength and aberration corrected multi-wavelength joint reconstructions using the colocalization regularization. Bottom-right:  Typical white light images of the 4 species studied here.

Figure 6 presents the spectral signatures of each type of bacteria with their respective error bars evaluated with the standard deviation. Three methods of reconstruction are compared: The spectra of Fig. 6A are evaluated using the uncorrected, independent reconstructions at each wavelength with a fixed focus evaluated at 431 nm, like the reconstructions presented in Fig. 4B. The spectra of Fig. 6B are evaluated with the same uncorrected method, but considering a separate estimation of the focus at each wavelength. The spectra of Fig. 6C are evaluated with our complete aberration corrected method, i.e. with an aberration corrected estimation of the focus, an aberration correction of the reconstructions and multi-wavelength joint reconstruction using the colocalization prior.

Examples of reconstructions using these 3 methods are presented in Fig. S1 in addition to Fig. 4.

**Table 1:** Amplitude of the spectral signatures calculated as the maximum minus the minimum of the transmittance (T) and OPD spectrum presented in Fig. 6 for the 3 reconstruction methods A, B and C and for the 4 bacteria species under study (S.A. Staphylococcus aureus, C.A. Corynebacterium auricosum, A.B. Acinetobacter baumannii, E.C. Escherichia coli).

	Gram +		Gram −	
	S.A.	C.A.	A.B	E.C
T (A)	0.26 ± 0.10	0.26 ± 0.07	0.06 ± 0.02	0.09 ± 0.03
T (B)	0.30 ± 0.10	0.30 ± 0.07	0.06 ± 0.02	0.10 ± 0.03
T (C)	0.34 ± 0.09	0.35 ± 0.05	0.09 ± 0.02	0.12 ± 0.02
OPD (A)	18.70 ± 8.91	18.80 ± 7.00	6.66 ± 2.35	7.96 ± 3.93
OPD (B)	21.94 ± 9.32	23.48 ± 9.83	7.50 ± 2.43	8.93 ± 4.00
OPD (C)	27.11 ± 9.28	32.48 ± 7.73	7.47 ± 2.91	9.79 ± 5.14

The dispersion is evaluated with the standard deviation on the bacteria. OPDs are expressed in nanometer.

As expected from the color reconstructions presented above, the transmittance spectral signatures show a lower minimum for Gram positive bacteria. However, this minimum is observed at 532 nm for both Gram types, whatever the reconstruction method. In diluted solution, the absorption peaks of crystal violet (that stains Gram positive bacteria) and safranin (that stains Gram negative bacteria) should be respectively, around 590 nm and 540 nm. During the staining procedure and at high concentrations inside the bacteria, it is well known that these values are likely to change. From our spectral analysis of the dyes inside bacteria, it appears that the location of the transmittance valley is less discriminating than its transmittance level. However, the reconstructions performed with our method reveal a more important difference between the spectral signatures of Gram positive and Gram negative types, both for transmittance and OPD spectral signatures. For more precise insights on these improvements, Table 1 presents the numerical values of the amplitude and dispersion of the spectral signatures for both transmittance and OPD.

The spectral signatures on the OPD show a valley and a peak for both types of bacteria. As the OPD spectral signature is supposed to mirror the refractive index dispersion, this kind of spectral evolution is consistent with the Kramers–Kronig relationships that describe the link between absorption and refraction properties of matter. Indeed, for a dye with one absorption band, it is common to observe a dispersion curve of the refractive index presenting a valley and a peak on both sides of the absorption maximum^37^. First, this result indicates that the OPD spectral signature we evaluated follows a trend with a clear and realistic physical meaning. Second, the OPD spectral analysis is an encouraging complementary way of characterizing the spectral signature of a stained biological object for classification purposes. Concerning the OPD spectral signatures, our reconstruction method clearly enhances the separation between the Gram positive and the Gram negative spectral signatures, in the same way as for transmittance spectral signatures. However, the maximum of OPD are clearly not centered on the same wavelengths for Gram positive and Gram negative bacteria. In these examples, the OPD signatures are thus spectrally more discriminant of the Gram type than it is with the transmittance signature. These results show that, with our corrected reconstruction methodology, the transmittance contrast between Gram positive and Gram negative bacteria is significantly enhanced. The OPD spectral signature is also improved and provides promising complementary spectral information to distinguish Gram positive and Gram negative bacteria, which, to our knowledge, is an original contribution in this context.

## Discussion

In this paper, we propose a multispectral reconstruction method in DIHM that consists in two steps. First, a calibration step is performed to estimate the focus positions as well as the aberrations of the holographic system for each wavelength and at different positions in the field of view. This calibration step uses a model-fitting approach on spherical calibration beads inserted in the sample and requires only one hologram per wavelength. This makes it possible to estimate the bead parameters along with the aberration parameters, which can also be performed with polydispersed objects. Second, the reconstruction step is performed to reconstruct the complex transmittance spectral stack from the same hologram spectral stack used for calibration. In a regularized inverse problems approach, this reconstruction step uses a precise multispectral forward model that accounts for the previously estimated aberrations. In this step, a single regularization that promotes colocalization of the objects on every spectral frame is used, which is a physically consistent hypothesis once the chromatic aberrations has been taken into account.

This unsupervised self-calibrated reconstruction method was applied to a specific case of Gram-stained bacteria. The results of the calibration step show that the estimated aberrations are consistent, continuous and repeatable and that the calibration step can be performed prior to the reconstruction on a calibration slide with beads alone, or, directly inside the sample, in which case the focus distance is also calibrated precisely. In the latter case, since spatially varying focus positions are considered, a possible tilt of the slide can be corrected.

After reconstructing data acquired on samples containing various types of Gram negative and Gram positive bacteria, we compared our method to similar reconstructions without aberration corrections and with independent processing at each wavelength. The results of these reconstructions were analyzed qualitatively in terms of contrast, color and morphology, but also quantitatively in terms of average transmittance and OPD spectral signatures of the bacteria. These analyses showed it is possible to perform a more complete (transmittance and phase), unbiased (by focus errors or aberrations), repeatable and multispectral microscopy characterization of Gram-stained blood smears. The results presented in this work demonstrate promising improvements to the capability of discrimination between two types of bacteria thanks to:

- better separation of the transmittance spectral signatures of Gram positive and Gram negative types,
- additional and complementary information provided by the OPD spectral signatures that are also easier to separate with our reconstructions,
- improvement in the evaluation of the sizes and shapes of bacteria.
- improvement in the separation of contiguous objects for better segmentation of individual objects.

Taken together, these results show that the influence of the experimental parameters (such as slide tilt, focusing errors and aberrations of the optical system) is reduced. The results thus pave the way for unsupervised automation and standardization of Gram analysis. Other similar biological analyses that use other staining procedures or even naturally colored microscopic objects like microalgae, could also benefit from our methodology.

Finally, in the field of holographic and quantitative phase microscopy, this study also demonstrates the non-negligible effects of certain residual aberrations and the way to estimate them precisely from only one hologram per wavelength. In a more general perspective, aberrations can have different origins (and are of varying importance): aberrations in the illumination beam, alignment errors, wrong coverslip thickness or wrong immersion medium, low cost objectives, non-planar optical windows, etc. Aberration problems may even concern certain lens-free configurations. Thus, it is important to note that in the general context of multivariate phase retrieval that exploits phase diversity, aberration correction methodologies such as the one presented here may be required.

## Methods

### Aberration calibration

The aberrations induced by the optical system are calibrated by fitting a forward model of the hologram to the data recorded with spherical beads inserted in the sample. In the absence of aberrations, the diffraction pattern ${\varvec{m}}_{Mie}$ of a spherical bead is accurately modeled by the Lorenz–Mie model^38^, which depends on the set of bead parameters $(x_{b},y_{b},z_{b},r_{b},n_{b})$, where $(x_{b},y_{b},z_{b})$ are the 3D coordinates, $r_{b}$ is the radius and $n_{b}$ is the refractive index of the bead. The Lorenz–Mie model has been successfully used to reconstruct spherical objects from holograms by fitting methods^20,39,40^ or, in a more general framework, by parametric IPA^12,19,41^.

In the presence of aberrations, the new image formation model of the diffraction pattern of the bead ${\varvec{m}}_{Mie}^{Ab}$ also depends on the aberration parameters ${\varvec{\alpha }}$ of the optical system. ${\varvec{{\varvec{\alpha }}}}=\left\{ {\varvec{\alpha }}_n^m \right\} _{(m,n) \in \mathbb {Z}^2}$ is a vector of aberration parameters referred to as Zernike coefficients in this article. These parameters are used to model the amplitude transfer function (ATF)^35^ of the optical system:

1 $$\begin{aligned} \tilde{{\varvec{h}}}^{Ab}(\nu _x,\nu _y,{\varvec{{\varvec{\alpha }}}})= {\left\{ \begin{array}{ll} e^{i\left[ \sum \limits _{n,m} {\varvec{\alpha }}_n^m {\varvec{Z}}_n^m(\nu _x,\nu _y)\right] } \quad if \quad \sqrt{\nu _x^2+\nu _y^2} < \frac{NA }{\lambda }\\ 0 \quad otherwise, \end{array}\right. } \end{aligned}$$

where ${\varvec{Z}}_n^m$ are the Zernike polynomials^22,23^ (see our previous study^12^ for details). $(\nu _x,\nu _y)$ are the spatial frequency coordinates, $NA$ is the numerical aperture of the objective and $\lambda$ is the illumination wavelength. $\frac{NA}{\lambda }$ is the cutoff frequency of the objective under coherent illumination. The new model is then expressed as follows:

2 $$\begin{aligned} {\varvec{m}}_{Mie} ^{Ab}({\varvec{\vartheta }})= \left| \mathscr {F}^{-1}\left[ \tilde{{\varvec{h}}}^{Ab}({\varvec{{\varvec{\alpha }}}}) \odot \widetilde{{\varvec{m}}}_{Mie} (z_b,r_b,n_b) \right] \right| ^2 , \end{aligned}$$

where $\odot$ is the Hadamard (element-wise) product. $\mathscr {F}^{-1}$ is the inverse Fourier transform operator and $\widetilde{{\varvec{m}}}_{Mie}$ is the Fourier transform of ${\varvec{m}}_{Mie}$. Note that for clarity, Fourier space coordinates and spatial coordinates are omitted in the equations when not required. Thus, the resulting model with aberrations depends on ${\varvec{\vartheta }}=\{x_{b},y_{b},z_{b},r_{b},n_{b},{\varvec{\alpha }}\}$.

Here, we describe the principle of calibration on a single bead, but our algorithm works with a multiple beads model. The set of Zernike coefficients ${\varvec{\alpha }}$ is estimated at the position of each bead $(x_{b},y_{b})$ since the aberrations vary within the field of view. Thus, the estimation of the axial position of the slide plane, as well as estimation of the pupil function is performed for each bead position by fitting the image formation model on the data, which consists in the minimization problem of the following cost function:

3 $$\begin{aligned} {\varvec{\vartheta }}^\dagger =\mathop {\textrm{argmin}}\limits _{{\varvec{\vartheta }}\in \mathbb {P}}\Vert {\varvec{d}}- {\varvec{m}}_{Mie} ^{Ab}({\varvec{\vartheta }})\Vert ^2_{{\varvec{W}}}, \end{aligned}$$

where ${\varvec{\vartheta }}^\dagger$ is the set of estimated parameters, $\mathbb {P}$ is the optimization domain of the parameters, and ${\varvec{d}}$ is the data, *i.e.* the hologram. Assuming the noise to be Gaussian, we chose a least square cost function weighted by ${\varvec{W}}$, the inverse of the covariance matrix of the noise. The covariance matrix of the noise can vary with the wavelength. In the case of stationary white Gaussian noise, $\forall \varvec{u}, \left\Vert \varvec{u}\right\Vert ^2_{{\varvec{W}}}=(1/\sigma ^2)\sum _{k}u_{k}^2$ where $\sigma ^2$ is the noise variance. Note that this data-fidelity term could be replaced by a robust cost function^42–44^ in the non-Gaussian case.

In these conditions, it has been previously showed that the parameters estimated with an aberration-corrected model are unbiased^11,12^. This is particularly interesting in the case of the $z_b$ and $r_b$ estimations of beads spread out in the field of view. They can then provide an accurate and unbiased estimation of the slide plane location and 3D orientation using a plane-fitting method similar to the one described in a previous study^8^.

### Multispectral regularized reconstruction

The multispectral reconstruction is performed using a regularized IPA. Since the objects are expected to be observed at the same positions whatever the wavelength, colocalization of the gradient of the real and imaginary part of the reconstructions can be added as an efficient and physical prior in the reconstructions^24^. A regularization term $\mathscr {R}$ that accounts for this prior can be written as:

4 $$\begin{aligned} \mathscr {R}({\varvec{t}})=\sum _k\sqrt{\sum _{\lambda \in \Lambda } \Vert {\varvec{\nabla }}_k \Re ({\varvec{t}}_\lambda )\Vert ^2+\Vert {\varvec{\nabla }}_k \Im ({\varvec{t}}_\lambda )\Vert ^2+\epsilon ^2 }, \end{aligned}$$

where $${\varvec{\nabla }}_k= \begin{bmatrix} \Delta ^x_k \\ \Delta ^y_k \end{bmatrix}$$ with $\Delta ^x_k$ (resp. $\Delta ^y_k$) a discrete gradient operator in the *x* (resp. *y*) direction, $\Lambda$ is the set of wavelengths, ${\varvec{t}}=\{{\varvec{t}}_\lambda \}$ is the stack of transmittances for all the wavelengths $\lambda$, and $\epsilon$ is a small-value scalar to ensure the differentiability of $\mathscr {R}$.

Thus, the regularized inverse problem can be stated as:

5 $$\begin{aligned} {\varvec{t}}^\dagger =\mathop {\textrm{argmin}}\limits _{{\varvec{t}}\in \mathbb {T}}\sum _{\lambda \in \Lambda }\Vert {\varvec{d}}_\lambda -{\varvec{m}}_\lambda ({\varvec{t}}_\lambda )\Vert ^2_{{\varvec{W}}_\lambda }+\mu \mathscr {R}({\varvec{t}}_\lambda ), \end{aligned}$$

where ${\varvec{m}}_\lambda ({\varvec{t}}_\lambda )= |{\varvec{h}}_{z,\lambda }^{RS} *{\varvec{t}}_\lambda |^2$ is the hologram formation model (without aberration correction) at wavelength $\lambda$ based on the Rayleigh–Sommerfeld kernel ${\varvec{h}}_{z,\lambda }^{RS}$^45^, $\mu$ is a regularization hyperparameter that balances the weight of priors versus the data fidelity term in the reconstruction and $\mathbb {T}$ is the optimization domain. In this study, we wanted to achieve the best accuracy on the reconstruction of the spectral signature of the bacteria in transmission and phase with our setup. Thus we used the 8 available wavelengths. However the proposed method is also valid with less wavelenghts (see Fig. S5).

### Tuning of the regularization hyperparameters

To automatically tune these hyperparameters, we use the fact that the bacteria and the beads are of similar sizes and induce similar phase shifts. Moreover, the reconstructed calibration beads are estimated by backpropagating a rigourous model (Lorenz–Mie). From the calibration step, a transmittance map of the bead can be simulated and considered as ground-truth transmittance ${\varvec{t}}^{GT }_{Beads}$ on the spatial support of the beads ${\varvec{W}}_{Beads}$ ($W_{Beads} (k,k)=1$ if the *k*-th pixel is within the bead support, 0 otherwise). The hyperparameters $\mu$ and $\epsilon$ can then be selected such that the reconstruction of the beads using the regularization be as close as the ground-truth obtained using model fitting. Accordingly, we propose an unsupervised method to tune these hyperparameters that consists in solving the following bi-level problem:

6 $$\begin{aligned} \begin{aligned}{}&\{\mu ^\dagger ,\epsilon ^\dagger \}=\mathop {\textrm{argmin}}\limits _{\{\mu ,\epsilon \} \in \mathbb {M}}\left\Vert {\varvec{t}}^{GT }_{Beads }-{\varvec{t}}^{\dagger }(\mu ,\epsilon )\right\Vert _{{\varvec{W}}_{Beads} }^2 \\&subject \,to: \\&{\varvec{t}}^\dagger (\mu ,\epsilon )=\sum _{\lambda \in \Lambda }\mathop {\textrm{argmin}}\limits _{{\varvec{t}}\in \mathbb {T}} \left\Vert {\varvec{d}}_\lambda -{\varvec{m}}_\lambda ({\varvec{t}}_\lambda ) \right\Vert _{{\varvec{W}}_\lambda }^2 + \mu \,\mathscr {R}_{\epsilon }({\varvec{t}}). \\ \end{aligned} \end{aligned}$$

Note that alternative unsupervised techniques, such as the Morozov’s discrepancy principle^46^, L-Curve^47^ or the minimization of the Stein Unbiased Risk Estimator (SURE)^48–50^ could also be considered to tune the regularization hyperparameters.

### Multispectral aberration-free reconstructions

First, for the reference wavelength $\lambda _{ref}$, the method we propose consists in estimating the parameters $(x_b,y_b,z_b,r_b,n_{b_{\lambda _{ref} }})$ of the beads and the 15 first Zernike coefficients ${\varvec{\alpha }}_{\lambda _{ref} }$. However, the piston coefficient $\alpha _0^0$ is set to 0 because the phase piston has no effect on the image formation model (intensity image formation model), $\alpha _1^{-1}$ and $\alpha _1^1$ are also set to 0 as they are responsible for lateral shifts in the positions of the beads (like *x* and *y* parameters). Finally, $\alpha _2^0$ is also set to zero since the defocus has already been estimated by parameter *z*. Thus, $\{\alpha _0^0,\alpha _1^{-1},\alpha _1^1,\alpha _2^0\}_{\lambda _{ref} }=0$. In this paper $\lambda _{ref} =431 \textrm{nm}$. Indeed, a study of the Cramér-Rao Lower Bounds showed that for the same amount of noise in the data, accuracy is better at smaller wavelengths (see Fig. S2). Note that smaller wavelengths also correspond to data with a better resolution.

Second, for the other wavelengths $\lambda \ne \lambda _{ref }$, we assume that the parameters $x_b,y_b,z_b,r_b$ are already known from the estimation at $\lambda _{ref}$, as they do not depend on the wavelength. However, we re-estimate the refractive index $n_b$ and all Zernike coefficients (except $\alpha _0^0$, which is equal to zero for every wavelength). Therefore, the estimation of $\{\alpha _1^{-1},\alpha _1^1,\alpha _2^0\}_{\lambda \ne \lambda _{ref} }$ accounts for lateral shifts and defocuses from the reference situation at $\lambda _{ref}$. Note that all other Zernike coefficients (spherical aberration, coma, astigmatism, etc.) are estimated independently for each wavelength in the process. These estimations were performed using an interior-point optimization algorithm and the model fitting of each bead for each wavelength required about 30 seconds on a Intel Core i7-13700F CPU 2.10 GHz with 16GBytes of RAM. Note that the aberrations estimations are not required for each image reconstruction as long as they have already been performed on one image.

It should be noted that the aberrations vary depending on the position in the field of view. Consequently, an interpolation of the ATF is needed. The interpolation method used here is based on a fast, state-of-the-art algorithm that computes an image formation model considering a spatially varying ATF^36^. It requires the knowledge of the ATF on regularly spaced positions in the field of view. To adapt it to our problem (the beads are randomly spaced) we just added a first step to interpolate the Zernike coefficients on a regular grid with the local polynomial regression method^51^. Estimating these aberrations on every bead position for each wavelength makes it possible to define an aberration-corrected forward model for regularized reconstruction:

7 $$\begin{aligned} {\varvec{m}}_\lambda ({\varvec{t}}_\lambda )=\left|\textit{P}_{{\varvec{X}}_b,{\varvec{Y}}_b,{\varvec{Z}}_b,{\varvec{A}}_{\lambda }}\left( {\varvec{t}}_\lambda \right) \right|^2\, \end{aligned}$$

where $\textit{P}$ computes the propagation at each pixel of ${\varvec{t}}_\lambda$ by considering the shift-variant aberrations using an interpolation of the ATF out of the parameters estimated in the calibration step (${\varvec{X}}_b$, ${\varvec{Y}}_b$, ${\varvec{Z}}_b$, ${\varvec{A}}_{\lambda }$ are the sets of all $x_b$, $y_b$, $z_b$, ${\varvec{\alpha }}_\lambda$). Note that if the ATF was invariant in the field of view and if the slide was not tilted, this propagator can be rewritten as a simple convolution: $\textit{P}_{{\varvec{X}}_b,{\varvec{Y}}_b,{\varvec{Z}}_b,{\varvec{A}}_{\lambda }}\left( {\varvec{t}}_\lambda \right) = {\varvec{h}}^{Ab}_{\lambda }*{\varvec{h}}_{z}^{RS}*{\varvec{t}}_\lambda$.

The image formation model is consequently more accurate because it considers aberrations that vary within the field of view and with $\lambda$. In addition, it ensures better fulfilment of the colocalization hypothesis at every wavelength. In the present study, the multispectral reconstructions were performed using the VMLMB algorithm^52^. Each reconstruction required 45 minutes on a NVDIA GeForce RTX 4070 Ti GPU. To speed up the reconstruction process, the reconstruction can be performed on small patches where the ATF is considered to be constant. Thus, the computation time of the reconstruction would be the same as that of an IPA reconstruction which does not consider the aberrations. However border effects on the patches are expected if the ATF varies too much between two patches.

**Figure 7:**
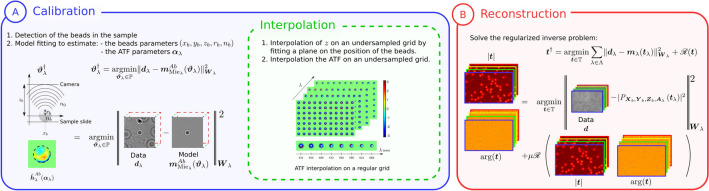
Schematic illustration of our DIHM regularized reconstruction methodology with the calibration of aberrations (**A**) and aberration corrected multispectral reconstructions (**B**).

### Visualization of the multi-wavelength reconstructions

In this study, a transmittance map and an OPD map is reconstructed for each of the 8 wavelengths. To display standard RGB images, the value of the transmittance for each wavelength is weighted by the spectral sensitivity of an RGB camera. The resulting color image should be comparable to standard white light images, except that it has been corrected for aberrations. False-color OPD maps are also created using the same principle. This color OPD map only represents the positive part of the OPD. Indeed, the biological objects have a positive OPD while beads have a negative OPD because the refractive index of silica is lower than the one of the immersion medium. Then, the beads do not appear in the reconstructed OPD maps. The same spectral sensitivity is used to weight the OPD reconstructions (the color OPD maps are displayed as if a color camera was sensitive to the OPD rather than to the intensity).

### Experimental setup

**Figure 8:**
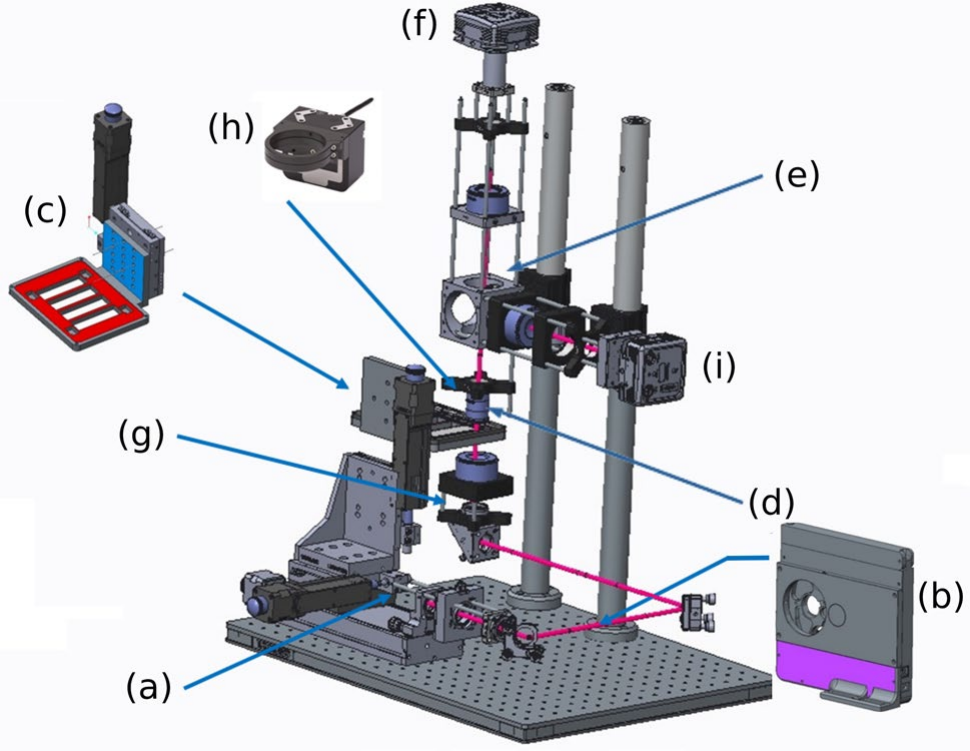
Illustration of the home-made automated DIHM setup used in this work with 8 wavelength partially coherent illumination: (**a**) fibered 8-LED light source, (**b**) filter wheel, (**c**) sample holder, (**d**) apochromatic objective, (**e**) beamsplitter T70/R30, (**f**) defocused monochrome camera, (**g**) incoherent light, (**h**) PIFOC piezo focus scanner, (**i**) in-focus color camera.

The DIHM setup with an 8-wavelength illumination is illustrated in Fig. 8. Since in-line holography does not require high coherence sources, an 8-LED fiber-coupled light source (from Mightex) was used for illumination. An additional set of 8 band-pass filters on a motorized filter wheel was used to further reduce the spectral width of the LED sources to 10 nm. This ensured a minimum coherence length of approximately $20\, \upmu \textrm{m}$ (at 431 nm), which is enough for in-line holography of micrometer-sized objects in an immersion medium. A Köhler illumination using a condenser lens with a focal length of 75 mm (doublet Thorlabs AC508-075-A) was set to ensure low-aperture illumination. Adjustable field and aperture diaphragms were set to control and optimize the spatial coherence of the DIHM illumination, to reduce stray light, and to ensure quasi-collimated illumination. In this setup, spatial coherence can be evaluated by the divergence of the illumination beam, which was around 10 mrad. After the sample, an infinity-corrected, apochromatic, oil-immersion microscope objective (Olympus MPLAPON 100XO) was used, with a numerical aperture of 1.4. A beamsplitter (70T/30R) then separated the imaging beam into two channels via two apochromatic tube lenses on two CMOS sensors. The channel (R) was equipped with a CMOS color sensor at the focus used for conventional incoherent white-light imaging and the other channel (T) was set out of focus and uses a monochrome sensor for sequential multispectral DIHM. The sensors were PL-D797 monochrome and color sensors from PixeLink, with $4.5 \, \upmu \textrm{m}$ pixel pitch and a 17.6 mm sensor diagonal. The defocus of the second sensor induced an average defocus of $10 \, \upmu \textrm{m}$ between the sample plane and the plane conjugated with the sensor.

### Sample preparation

Each blood culture bottle was inoculated with 10 mL of blood from a healthy donor and $200 \, \upmu \textrm{L}$ of bacterial suspension (colonies from Petri plate previously diluted in 0.85% NaCl solution) to simulate a bacteremia level of  5–20 CFU per bottle. Blood culture bottles were incubated in the appropriate automated blood culture instrument. Next, Gram-stained blood smears were obtained from positive blood culture after 24 h of incubation and mixed with calibrated silica beads of $1 \, \upmu \textrm{m}$-diameter (from SIGMA-ALDRICH). The refractive index of silica is 1.46 at 532 nm. Samples were spread out on the microscope slide using a semi-automated standard method developed at bioMérieux, then fixed in ethanol to avoid heating that degrades the red blood cells, and stained using a standard method, using the bioMérieux Gram staining system (PREVICOLOR). The resulting objects immobilized on the surface of the slide are pink (Gram negative) or purple (Gram positive). After drying, the slide was placed in the sample holder of the microscope in an oil immersion medium ($n_0= 1.519$).

### Supplementary Information

Supplementary Figures.

## Data Availability

The datasets used and analysed during the current study available from the corresponding author on reasonable request. An example of multispectral acquisition is provided in the supplementary information (Fig. S3).
