# Supplementary material for: Multispectral in-line hologram reconstruction with aberration compensation applied to Gram-stained bacteria microscopy
Source: Sci Rep. 2023 Sep 2;13:14437. doi: 10.1038/s41598-023-41079-4 (PMC10475072; doi:10.1038/s41598-023-41079-4)
Supplement: Supplementary file 1 — Supplementary Figures. [file 41598_2023_41079_MOESM1_ESM.pdf]

Multispectral in-line hologram reconstruction with aberration compensation applied to Gram-stained bacteria microscopy

Supplementary information

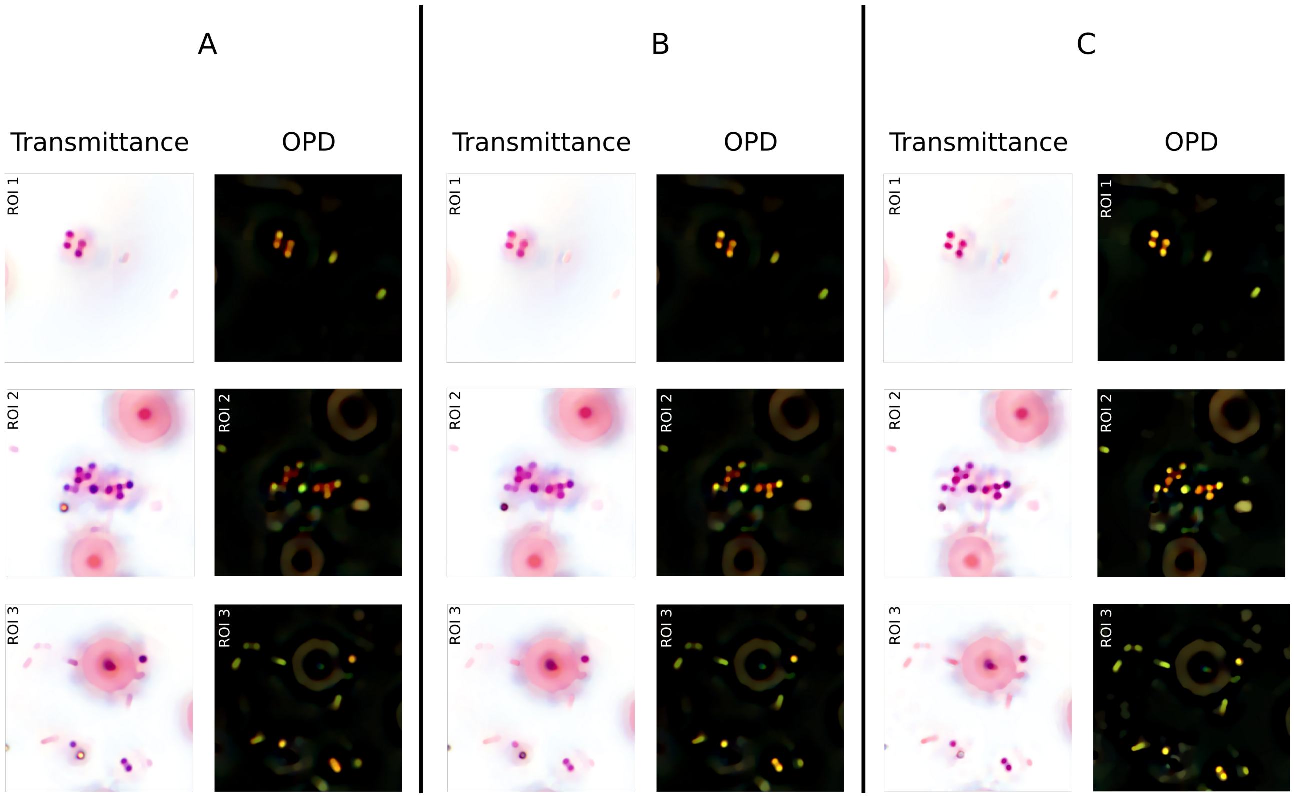

Figure S1: . Illustration of transmittance and OPD color reconstructions of a sample containing two types of bacteria (Gram positive Staphylococcus aureus and Gram negative Escherichia coli).

(A) Regularized reconstructions reconstructed independently at each λ , with no correction of aberrations and at a fixed focus estimated at 431nm. (B) Regularized reconstructions reconstructed independently at each λ , with correction of defocus aberrations by re-estimating z at each wavelength. (C) Multi-wavelength joint reconstructions with aberration correction and with the colocalization prior

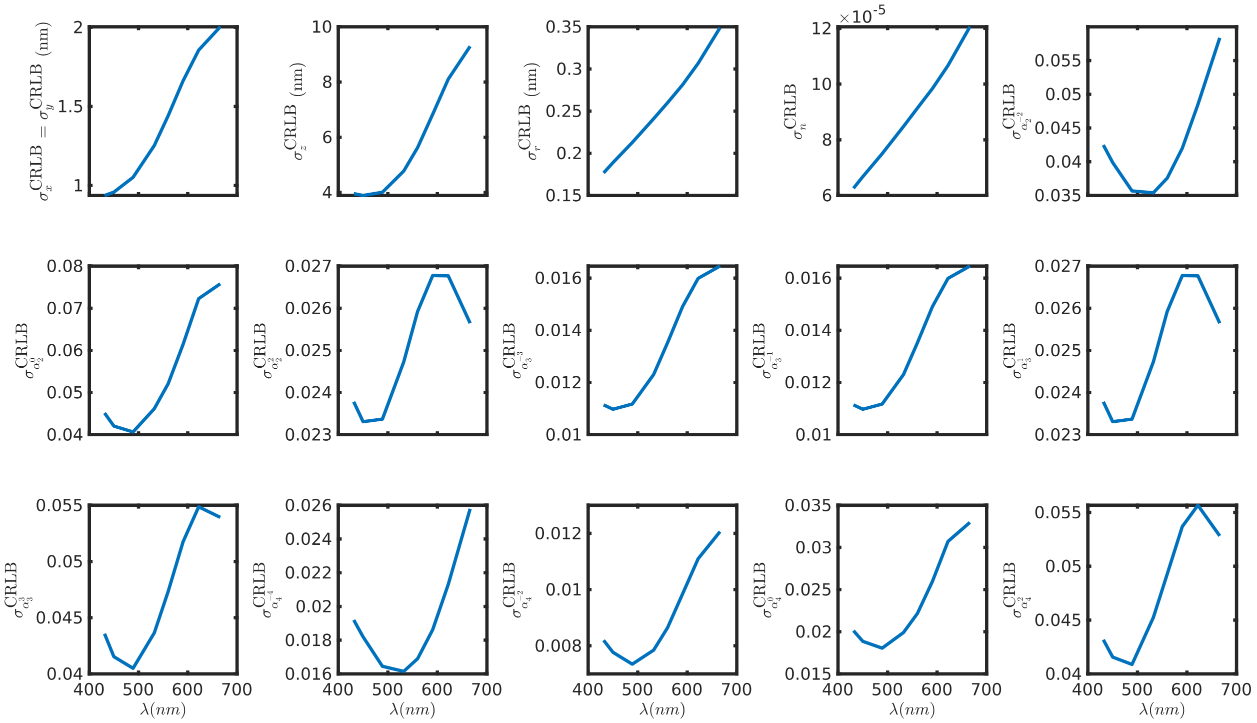

Figure S2: Evolution of the Cramér-Rao Lower Bound (CRLB) as a function of the wavelength. These CRLB have been computed for **α**=**0**.

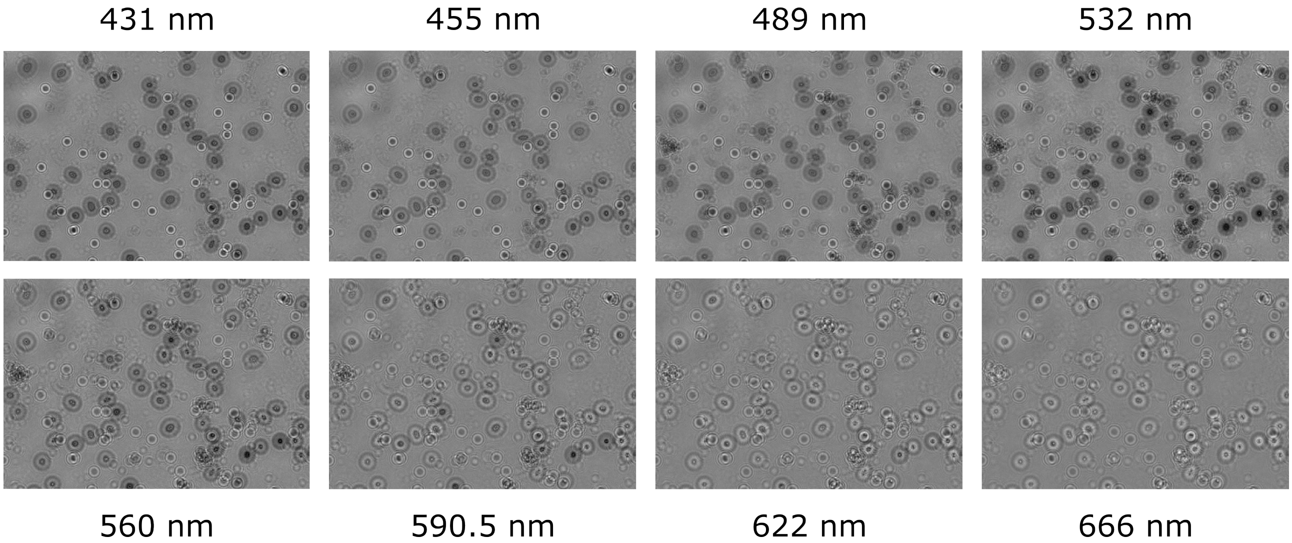

Figure S3: Example of multispectral acquisitions

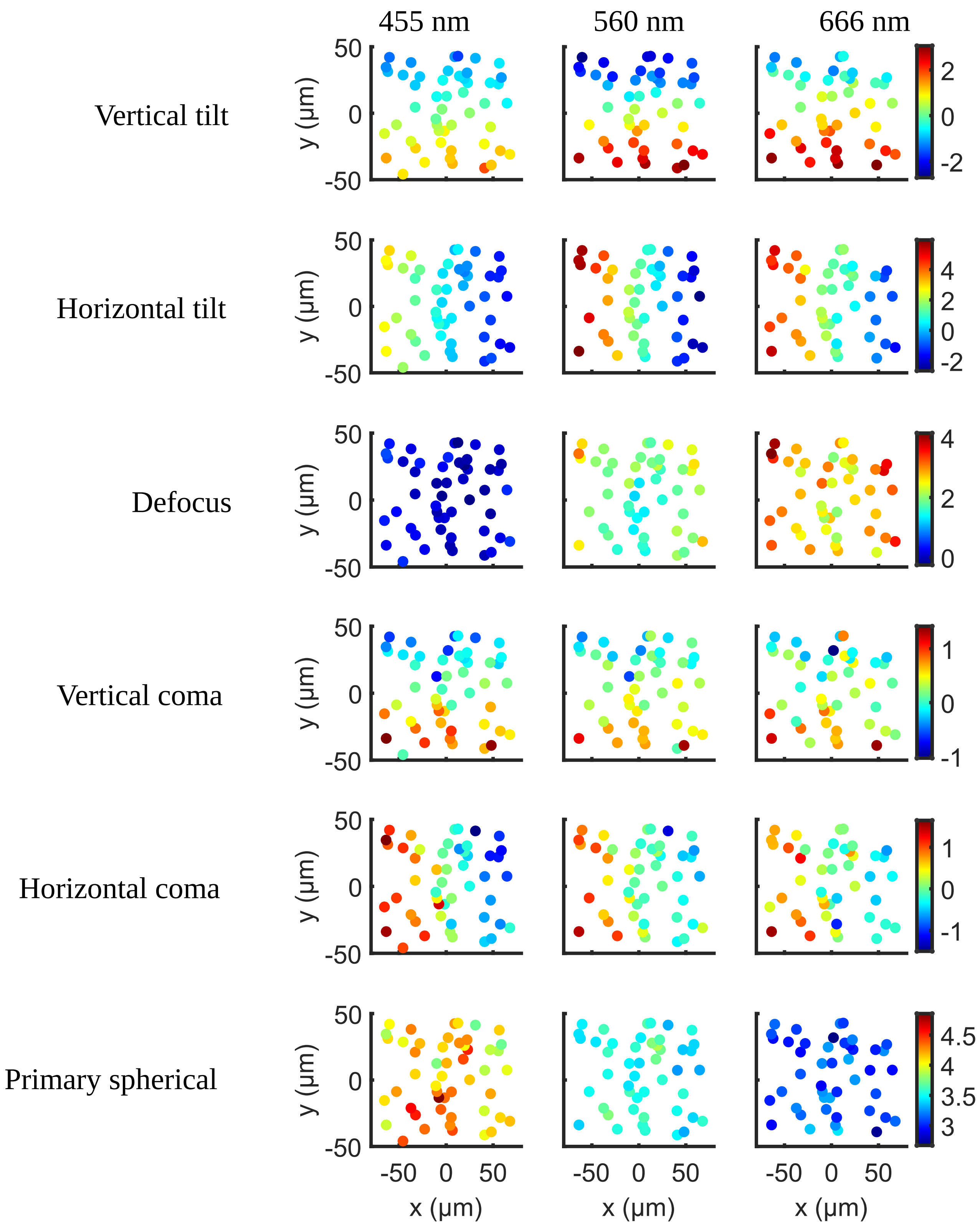

Figure S4: Evolution of the Zernike coefficients of the main aberrations in the field of view at three wavelengths. These estimated coefficient maps are used to compute Figure 2 and Figure 3.

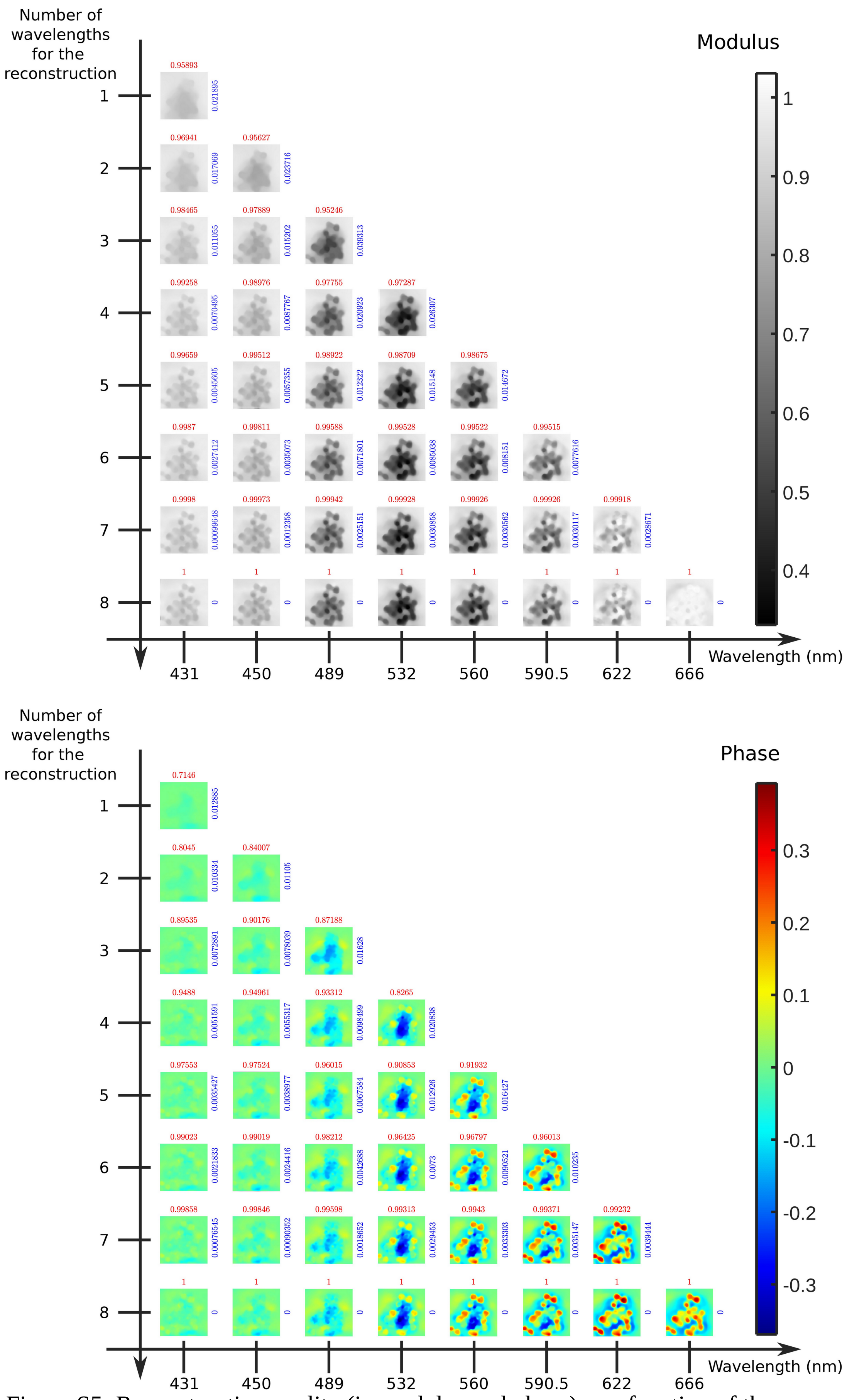

Figure S5: Reconstruction quality (in modulus and phase) as a function of the number of wavelengths used for the reconstruction. The Structural SIMilarity (SSIM) between the reconstruction and the considered ground truth (reconstruction with 8 wavelengths) is provided in red while the Root Mean Square Error (RMSE) is provided in blue. This figure highlights the benefit of phase diversity in the quality of our reconstruction.
